# A Comparative Study of 2-Hour Interface Pressure in Different Angles of Laterally Inclined, Supine, and Fowler’s Position

**DOI:** 10.3390/ijerph18199992

**Published:** 2021-09-23

**Authors:** Soo-Yeon Kim, Yong-Soon Shin

**Affiliations:** 1 Department of Nursing, Daegu Haany University, Gyeongbuk 38610, Korea; sooyeonkim@dhu.ac.kr; 2 College of Nursing, Hanyang University, Seoul 04763, Korea

**Keywords:** pressure ulcers, position change interval, different angles

## Abstract

Insufficient research exists for position change intervals to eradicate pressure ulcers. We tried to provide evidence for the position change interval by comparing peak pressure, risk area ratio, and the time to reach 30 mmHg and 60 mmHg, and presented this in detail, according to the angle in the three positions. The study conducted RCTs on a total of 64 healthy adults. For two hours, interface pressure measurements were compared with 30° and 90° tilting at the inclined, 0° and 45° head-of-bed (HOB) elevation at the supine, and 30° and 45° HOB elevation at the Fowler’s position. The peak pressure on 30° tilting remained less than 60 mmHg for 2 h, unlike 90° tilting. To reach 60 mmHg took 78.18 min at 30° tilting, within 30 min at the 30° supine, 30° and 45° at the Fowler’s position, and 39.55 min at 0° supine. The pressure difference according to the angles was only significant at 30° and 90° tilting, with no difference in the other groups. To prevent pressure ulcers, position changes are required every 2 h in the 30° tilting position, every 1.5 to 2 h at 0° supine, and at least every 1.5 h for all the other positions.

## 1. Introduction

Pressure ulcers refer to local tissue damage resulting from external forces such as continuous pressure, shear force, and friction [1], and are an important part of nursing care in terms of maintaining skin integrity [2]. Pressure ulcers do cause discomfort or pain owing to ischemia and tissue damage [3], and physical problems that cause complications, lead to poor clinical outcomes, psychological, and social problems caused by increased health care costs [4]. Since various problems are connected, a preventive approach is essential.

Accordingly, the clinical field uses the occurrence of pressure ulcers as an indicator of quality of care [5]. Therefore, efforts are made to prevent the occurrence of pressure ulcers through guidelines and research [4]. Pressure ulcers are caused by a variety of causes and depend on the individual condition of the patient. Therefore, pressure ulcer preventive nursing interventions suitable for individual subjects need to be provided, and such arbitration must be based on scientific evidence.

Practical guidelines for preventing pressure ulcers provide risk factors related to pressure ulcers and methods for preventing and managing pressure ulcers, including skin assessment, changing position, and the use of support surfaces. Although these guidelines are widely used clinically, recommendations on body posture changes are less specific and there is still insufficient literature to support the recommendations [6,7,8]. Some guidelines for preventing pressure ulcers provide recommendations for specific postures, such as 30° tilt and 30° or less head elevation [9,10,11,12]. However, conflicts with this recommendation, such as the unavoidable need to adopt a specific position for therapeutic purposes, may lead to confusion in clinical decision-making. This is because specific details such as the allowable range of head angle and posture maintenance time were not sufficiently presented [13].

Additionally, the level of evidence for repositioning recommendations is very low, or it is stated that there is insufficient evidence to compare between prophylactic interventions [9,10,11,12]. In each guideline, recommendations for position change were based on two or more non-RCTs in WOCN (2016) guidelines, expert opinions/institutional clinical experiences in RANO (2016) guidelines, and RCTs with very large limitations, observational studies, and case series in KCE (2012) guidelines. Specifically, the position for the prevention of bedsores in the guidelines used in clinical practice to date is based on insufficient evidence, and the conclusions about obvious clinical benefits and risks have not yet been reached. Furthermore, in the recent systematic review studies conducted in 2019 [14] and 2020 [15], it was difficult to find a concrete conclusion on the effect of pressure ulcers prevention according to position, which necessitates additional research to present the appropriate evidence for the preventive position.

## 2. Related Works

Pressure load time and pressure levels are important factors contributing to the occurrence of pressure ulcers [16]. For the redistribution of pressure before vascular occlusion occurs, the body surface pressure level must be understood, and the repositioning schedule or posture should be determined according to the critical point of pressure. However, even in a recently published study, the pressure for each position was measured for a brief period of about 10 min, so there is a limit to its clinical application [17]. Until now, numerous studies have reported the critical point of pressure ranging from 30 to 80 mmHg [18,19,20], but studies confirming the time to reach this critical point are insufficient, which necessitates an empirical study.

Taken together, the bedsore prevention guidelines lack quantitatively the studies designed to confirm strong causality such as RCT, and the studies reflected in the guidelines do not fully reflect the results owing to qualitative and methodological limitations (e.g., considerable risk of bias). As such, well-designed RCT studies must be conducted. In addition, a study measuring the time to reach the critical pressure suggested in previous studies by identifying the cumulative pressure loaded in each position up to the 2-hour starting point [21] is used as an empirical basis rather than the short-time measured pressure needed.

Therefore, we attempted to measure cumulative pressure for two hours at each body position with different angles and provide the appropriate position change interval for pressure injury prevention.

## 3. Materials and Methods

### 3.1. Aim

In this study, the peak pressure, risk area ratio, and time to reach pressure ulcer threshold pressure were compared in each group for 2 h at 30-min intervals. The three positions are laterally inclined (30° vs. 90°), supine with head-of-bed (HOB) elevation (0° HOB vs. 30° HOB elevation), and Fowler’s with 30° leg elevation (30° HOB elevation vs. 45° HOB elevation). The specific objectives were as follows.

- To identify the peak pressure of bony prominence according to each group in each position.
- To calculate and compare the risk area ratio for each position.
- To compare the time to reach the interface pressure of 60 mmHg between the two groups in each position.

### 3.2. Study Design

This study is the RCT to identify the interface pressure for each part of the body according to the type of position for 2 h. This study is divided into three trials: the laterally inclined, supine, and Fowler’s position, and compares the pressure change over time in the experimental and control groups within each trial, respectively. The angles for each position were divided into an experimental group and a control group based on the previous study. For the laterally inclined position, 30° tilting was used for the experimental group and 90° tilting for the comparison group [22]. For the supine position, 0° HOB was used for the experimental group, and the 30° HOB elevation was used for the comparison group [23]. For the Fowler’s position (supine combined with 30° leg elevation), 30° HOB elevation was used for the experimental group and the 45° HOB elevation for the comparison group [24,25]. For randomization, the Internet Randomization Service of Sealed Envelops™ was used, the group was set to two and the block size was set to 4 to generate a random list, and based on this, subjects were randomly assigned to an experimental group and a control group, respectively. The order of assignment was kept concealed by confirming the results of the assignment immediately before the experiment. Random assignment and provision of experimental intervention were performed independently by the principal investigator and the researcher, respectively.

### 3.3. Study Participants

This study was conducted on healthy adults who had little difficulty in taking an immobile position for 2 h. Although healthy young adults may differ from patient conditions, we have tried to provide basic data for healthy adults. Participants were recruited using the university’s online bulletin board. Exclusion criteria were subjects with skin damage, underlying diseases such as musculoskeletal disorders, peripheral circulation disorders, mental disorders, and those having difficulty maintaining the position for 2 h. Using G*power 3.1.9.7, the number of subjects required for repeated measures ANOVA was calculated. For the effect size, a medium size of 0.25 was used because there was no previous study with a similar design. A significance level of 0.05, a power of 0.80, two groups, and five measurements were input for the “number of measurements.” The number of subjects was calculated to be 11 for each group and 22 for each position (laterally inclined, supine, and Fowler’s). A total of 66 people were recruited, of which data of 64 people were included in the analysis, excluding two in which body interface pressure decreased rapidly owing to movement.

### 3.4. Variables and Measurements

#### 3.4.1. Interface Pressure

The contact interface pressure is the pressure applied to the patient’s skin, which is the pressure measured between the patient’s body surface and the bed. In this study, the interface pressure was calculated using two variables that indicated the strength and redistribution of pressure. The strength of pressure was identified using the peak pressure [26,27], and the redistribution of pressure was confirmed using the risk area ratio [28]. Using the XSENSOR X3 PX100 system (ForeSite SS Mattress System, XSENSOR Technology, Calgary, AB, Canada), we measured interface pressures in the subjects’ bony prominence sites. A flexible and thin 81.2 cm × 203.2 cm mattress sensor pad (PX100:26.64.01, XSENSOR Technology, Calgary, AB, Canada) can export pressure data sensed in contact with the body to a computer program running the X3 Pro (XSENSOR Technology, Calgary, AB, Canada) software. The sensor pad must be large enough to measure the pressure from the whole body in contact with the bed in any position on the bed. The entire sensor pad can measure pressure from 1296 sensors, 12.5 mm in size, with a measurement range of 10–200 mmHg and an accuracy rate of ±5%. The real-time numerical data could be retrieved into Microsoft Excel 2016 (Microsoft Corp., New York, NY, USA), and the pressure data is stored in 3600 frames for 2 h. For the pressure data, 5 min of data were extracted every 30 min, 60 min, 90 min, and 120 min, and the average value was calculated and used for analysis. The interface pressure was measured on a standard hospital mattress rather than on a support surface so that it was measured under the same conditions from all subjects. BMI (body mass index), which is one of the confounding variables that can affect the interface pressure, was not controlled when the subjects were selected, but it was controlled by a statistical method by comparing the BMI between groups.

#### 3.4.2. Peak Pressure

The peak pressure is the highest pressure among the pressures measured from each sensor. It refers to the peak pressure of the bone protrusion. When the subject took a position in the bed, the contact area with the bony prominence site was marked on the sensor pad using a sticker (for example, the shoulder and the greater trochanter in the lateral position; the scapula and sacrum are in the supine position or in the Fowler’s position). The position of the marked sensor can be checked in the pressure mapping, which is displayed visually in the X3 Pro program so that the pressure in that area can be measured.

#### 3.4.3. Risk Area Ratio

The risk area ratio indicates whether the pressure is evenly distributed and was used in previous studies [28] as an indicator of the risk of bedsores. The risk area ratio was calculated by calculating the ratio of the area measured with interface pressures of above 30 mmHg [29] and 60 mmHg [18], known as the interface pressure of the risk of pressure ulcers in previous studies, to the total area where pressure was measured. Thus, it refers to a percentage calculated by using the total number of sensors measuring pressure as the denominator and the number of sensors measuring 30 mmHg or 60 mmHg or more as the numerator. The threshold for pressure sores is reported in numerous studies, but the most commonly cited values are 30 mmHg [29] and 60 mmHg [18]. Therefore, we calculated the risk area ratio based on these two thresholds. The higher this value, the higher the risk of pressure ulcers.

### 3.5. Procedures and Data Collection

The principal investigator performed the randomization generation, and the research assistant was responsible for measuring the contact surface pressure according to the assigned group to maintain the concealment of randomization. Owing to the nature of the experimental study, it was not possible to keep the participants blind, but the researcher who did not know the assignment performed the analysis and maintained a single-blind procedure.

All participants were asked to lie in the prone position on a standard hospital mattress for 20 min, rest, and then hold the position corresponding to each group for two hours. The laterally inclined position group was to take a 30° or 90° tilting position according to the assignment. According to the assignment, the supine group was asked to take a supine position with a head elevation of 0° or 30°. The Fowler’s position group was asked to take a head elevation of 30° or 45° depending on the assignment. The baseline contact interface pressure was measured 5 min after taking the assigned position, and the contact interface pressure was measured after 30 min, 60 min, 90 min, and 120 min. For accurate measurement, study participants had to remain immobile for 2 h. Furthermore, no additional interventions were provided to the participants. Data were collected from November 2016 to June 2019.

### 3.6. Data Analysis

This study data were analyzed by dividing the three groups into position, supine, and Fowler’s, and the experimental and control groups were compared within each group. As a result of testing for normality with Shapiro–Wilk in this study, parametric analysis was performed on variables satisfying normality and nonparametric analysis of variables that did not satisfy normality. Continuous variables were summarized as the mean and standard deviation or median and IQR, and categorical variables were summarized as real numbers and percentages. The homogeneity test between the experimental and control group was analyzed by the Chi-square and independent t-test. Differences in peak pressure and risk area ratio (≥30 mmHg) between groups over time were analyzed by Repeated Measure ANOVA. Sphericity was assessed during Repeated Measure ANOVA analysis, and the Greenhouse Geisser’s modified test statistic was applied when the sphericity assumption was not satisfied in Mauchly’s test. In addition, when a significant difference was confirmed in the groups in Repeated Measure ANOVA, the difference between the groups was also confirmed using the *t*-test. The difference in risk area ratio (≥60 mmHg) between groups over time was analyzed by the Friedman test, and the difference in contact pressure between groups at each measurement point was analyzed by the Mann–Whitney U test. The time until the maximum pressure reached 60 mmHg was obtained using the Kaplan–Meier estimation method, and the results were presented as a cumulative survival function. The difference between the two groups was confirmed by log-rank (Mantel–Cox). For statistical analysis, the SPSS 21.0 WIN version(SPSS Inc., Chicago, IL, USA) was used, and the significance level of all analyses was 0.05.

### 3.7. Ethical Considerations

This study was conducted after obtaining approval from the Institutional Review Board (IRB) of Hanyang University. Participants were recruited through publicity within the university. The research was conducted having obtained written consent after the researcher had explained the research.

## 4. Results

### 4.1. General Characteristics of Participants, Verification of Homogeneity between Groups

The characteristics of the participants are shown in Table 1. As a result of verifying the homogeneity of the general characteristics of the experimental and control group in the three groups in the laterally inclined, supine, and Fowler’s position groups, gender, age, and body mass index (BMI) were all homogeneous in the group divided by position.

### 4.2. Peak Pressure of Contact Surface and Risk Area Ratio (30° vs. 90° Laterally Inclined Position)

**Figure 1:**
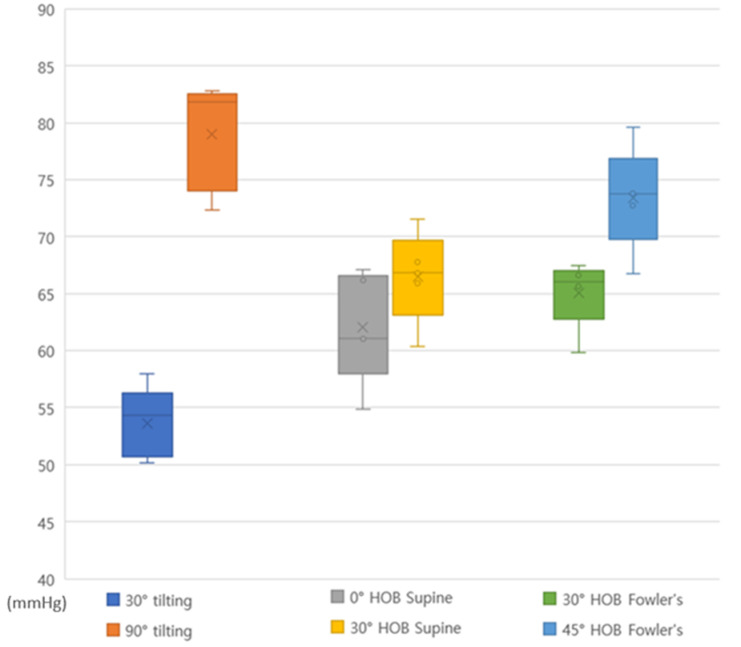
Interface peak pressure over 2 h by position (30° vs. 90° tilting; 0° vs. 30° HOB supine; 30° vs. 45° HOB Fowler’s position).

Table 2 and Figure 1 show the pressure change for 2 h observed at 30° and 90° laterally inclined positions. The peak pressure was 42.82–46.93 mmHg in the shoulder region and 47.45–50.85 mmHg in the greater trochanter region at 30° tilting positioning. In the 90° tilting positioning, it was 55.82–62.41 mmHg in the shoulder region and 69.54–79.15 mmHg in the greater trochanter region. At 90° tilting positioning, the greater trochanter was higher than 60 mmHg at all time points.

As a result of ANOVA, there was a difference between the 30° and 90° tilting positioning groups in the shoulder region (F = 9.083, *p* = 0.007), and the difference with time was also significant (F = 3.631, *p* = 0.029). In the greater trochanter, a difference between the 30° and 90° tilting positioning groups (F = 6.350, *p* = 0.002) was found, and the difference with time was also significant (F = 13.446, *p* = 0.002).

Based on the critical pressure of 30 mmHg for the risk of pressure ulcers, a difference showed in the risk area ratio between the 30°and 90° tilting positioning groups (F = 16.72, *p* = 0.001) with the risk area ratio within the group over time (pressure ulcer risk pressure 30 mmHg or more area ratio) as significant (F = 27.03, *p* < 0.001). Additionally, there was an interaction between time and group (*p* = 0.26), which showed a difference in the change in the risk area ratio over time in the 30° and 90° tilting positioning groups, respectively.

Based on the critical pressure of 60 mmHg for the risk of pressure ulcers, no difference with time in both the 30° and 90° tilting of the area of the dangerous pressure was found. When each group was compared per time, the 90° tilting position at all time points from baseline to 120 min showed a significantly higher risk area ratio than the 30° tilting position. Thus, in the positioning, the area that received pressure of 60 mmHg or more did not widen over time in both groups, and the 90° tilting position received more than 60 mmHg of pressure in a wider area than the 30° tilting position depending on the angle.

**Figure A1:**
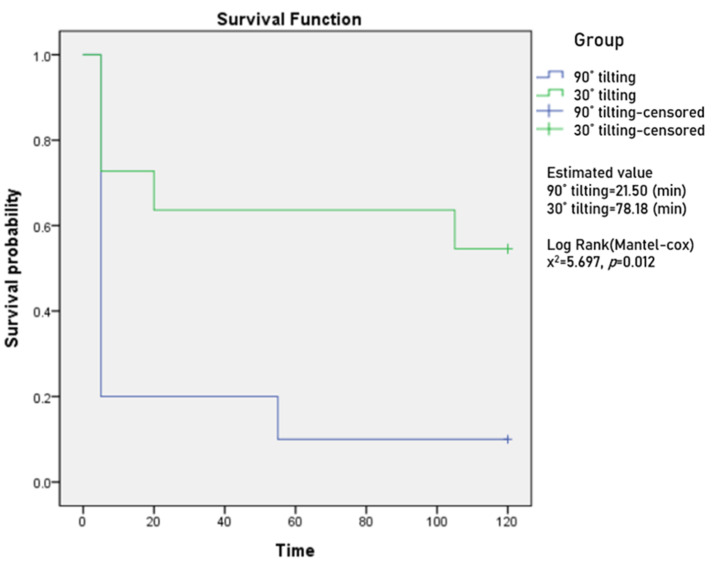
Estimates of survival time for the laterally inclined position group.

Among the total interface pressure, the average estimated time for the peak pressure to reach 60 mmHg was 78.18 min (SE 15.92) for 30° tilting (95% CI, 46.98–109.38), and 21.50 min (SE 11.40) for 90° tilting position (95% CI, 0.00–43.85) (Figure A1). There was a significant difference according to the angle in the laterally inclined position (*p* = 0.017).

### 4.3. Peak Pressure of Contact Surface and Risk Area Ratio (0° vs. 30° HOB Elevation Supine Position)

Table 3 and Figure 1 show the pressure change observed in the 0° HOB and 30° HOB elevation supine position for 2 h. In the 0° HOB elevation supine position, the peak pressure was 41.56–50.46 mmHg in the scapula region and 52.41–65.05 mmHg in the sacrum region. The 30° HOB elevation supine position was found to be 46.23–1.03 mmHg in the scapula region and 56.47–70.03 mmHg in the sacrum. The highest pressure was observed in the sacrum in both 30° and 90° HOB elevation supine positions.

As a result of the ANOVA, no difference between the 0° and 30° HOB elevation supine groups in the scapula region (F = 0.246, *p* = 0.625) was observed, and the difference with time was not significant (F = 2.807, *p* = 0.063). In the sacrum region, no difference between the 0° and 30° HOB elevation supine groups (F = 2.147, *p* = 0.158) was found, but the difference with time was significant (F = 30.672, *p* < 0.001).

Also, no difference was found in the risk pressure area ratio between the 0° and 30° HOB elevation supine groups based on the critical pressure of 30 mmHg for the risk of pressure ulcers (F = 1.002, *p* = 0.329). The risk area ratio within the group over time (pressure ulcer risk pressure 30 mmHg or more area ratio) was significant (F = 0.716, *p* < 0.001).

Based on the critical pressure of 60 mmHg for the risk of pressure ulcers, the risk area ratio showed a difference in time in both the 0° HOB (*p* = 0.03) and 30° HOB (*p* < 0.001) elevation supine positions. When comparing groups over time, 30 min (*p* = 0.032) and 60 min (*p* = 0.032), 90 min (*p* = 0.036) for the risk area ratio of 30° HOB was significantly higher than that of 0° HOB elevation supine.

**Figure A2:**
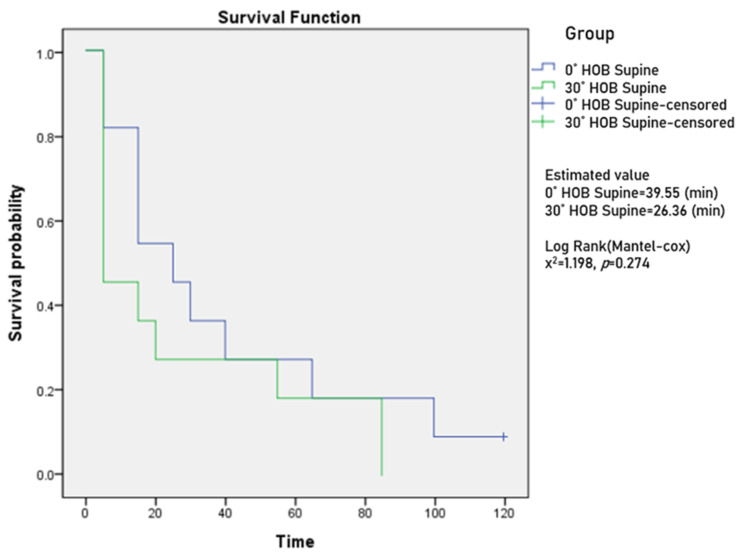
Estimates of survival time for the supine position group.

Among the total interface pressure, the average estimated time for the peak pressure to reach 60 mmHg among total interface pressures was 39.55 min (SE 11.24) in the 0° HOB elevation supine position (95% CI, 17.53–61.57), and 26.36 min (SE 9.82) at 30° HOB elevation supine (95% CI, 7.12–45.61) (Figure A2). There was no significant difference according to the angle in the supine position (*p* = 0.274).

### 4.4. Peak Pressure of Contact Surface and Risk Area Ratio (30° vs. 45° HOB Elevation Fowler’s Position)

Table 4 & Figure 1 show the pressure change for 2 h observed in the 30° and 45° HOB elevation Fowler’s position. The peak pressure was 50.66–60.10 mmHg at the scapula and 55.19–58.06 mmHg at the sacrum in 30° HOB elevation Fowler’s position, 51.32–58.26 mmHg at the scapula and 60.65–70.24 mmHg at the sacrum in 45° HOB elevation Fowler’s position. The peak pressure was less than 60 mmHg at all time points except for the scapula at 120 min in the 30° HOB elevation Fowler’s position, and the sacrum in the 45° HOB elevation Fowler’s position exceeded 60 mmHg at all time points.

As a result of the ANOVA, no difference between the 30° and 45° HOB elevation Fowler’s position groups in the scapula region (F = 0.002, *p* = 0.964) was observed, and the difference with time was significant (F = 5.404, *p* = 0.001). In the sacrum region, there was no difference between the 30° and 45° HOB elevation Fowler’s position groups (F = 1.244, *p* = 0.279), but the difference with time was significant (F = 7.954, *p* = 0.002).

There was no difference in the risk pressure area ratio between the 30° and 45° HOB elevation Fowler’s position groups (F = 0.716, *p* = 0.408) based on the critical pressure of 30 mmHg for the risk of pressure ulcer occurrence, and the risk area ratio within the group over time (pressure ulcer risk pressure 30 mmHg or more area ratio) was significant (F = 20.020, *p* < 0.001).

Based on the critical pressure of 60 mmHg for the risk of pressure ulcers, the area ratio of the dangerous pressure did not differ according to the time flow in both the 30° and 45° HOB elevation Fowler’s position. There was no significant difference even when comparing each group per hour.

**Figure A3:**
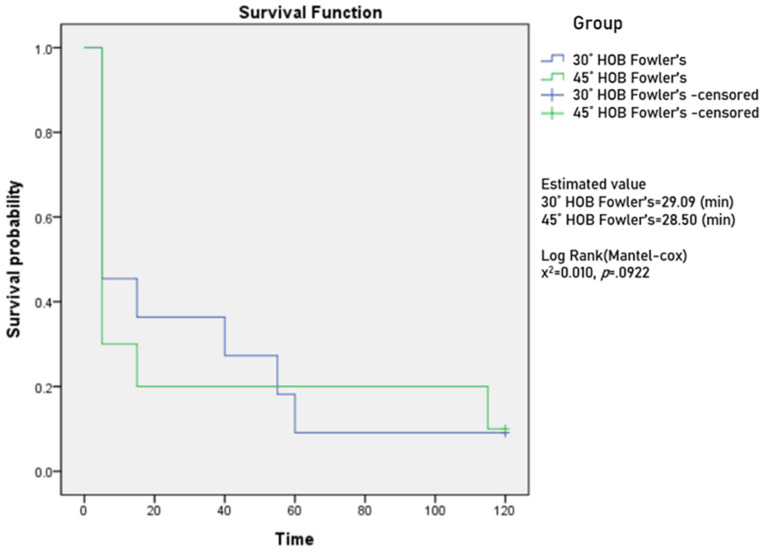
Estimates of survival time for the Fowler’s position group.

Among the total interface pressure, the average estimated time for the peak pressure to reach 60 mmHg was 29.09 min (SE 10.64) for 30° HOB elevation in the Fowler’s position (95% CI, 8.42–49.94), and 28.50 min (SE 14.11) for 45° HOB elevation in the Fowler’s position (95% CI, 0.85–56.15) (Figure A3). No significant difference according to the angle in the Fowler’s position (*p* = 0.922) was present.

## 5. Discussion

This study aimed to identify changes in interface pressure over time in the laterally inclined, supine, and Fowler’s positions in healthy adults, to present evidence for a position in the guidelines for preventing pressure ulcers, and to prepare specific recommendations for a position change. This study was conducted in the immobile position for 2 h after the participants took the corresponding position, and the peak pressure and peak pressure in the bony prominence were measured for each time period. Additionally, to calculate the area with a substantial risk of pressure ulcers, the ratio of pressure ulcer risk area among the entire body surface was compared between groups based on the critical values of pressure ulcer generation, 30 mmHg and 60 mmHg, respectively. Finally, according to the group by position, the time point at which a high pressure of 60 mmHg or more was generated was observed in units of 5 min to present evidence for the position change interval. Traditionally, the threshold for pressure ulcers is 32 mmHg owing to capillary occlusion [30]. However, a study involving humans for an extended period reported higher pressure [20]; thus, we compared the time of reaching the standard between groups. Therefore, we used 60 mmHg as the risk criterion for pressure ulcers and compared the time to reach this criterion between groups. By synthesizing the results of the above study, this study compares the evidence for position for the prevention of pressure ulcers presented in previous studies and guidelines and discusses the clinical application direction.

First, looking at the laterally inclined position, the measurements were significantly higher at 90° tilting than at 30° tilting for the two hours, and the highest pressure measured on the entire body interface was higher than 60 mmHg from the start. Pressure applied to the protrusion is known to be a strong cause of pressure ulcers among numerous factors [31]. The 90° tilting position can be seen as the average pressure on the contact surface that is higher than that of the other position because the position of the shoulder and greater trochanter are more protruding. This is a result consistent with the existing guidelines for avoiding the 90° tilting positioning and recommending the 30° tilting position [32]. However, even if a 30° tilting position is recommended, a recent study considering BMI showed higher pressure in obese women than overweight, normal, and underweight people in a 30° tilting position [17]. Therefore, even with a 30° tilting position, the skin condition of these patients should be assessed frequently.

When 30 mmHg was set as the critical pressure standard for pressure ulcers to occur, the risk area ratio showed a significant difference between 30° and 90° tilting positions as time passed. In particular, in taking the laterally inclined position for 2 h, the risk area ratio was about 30% and 40% at the 30° and 90° tilting positions respectively, confirming that the risk of pressure ulcers was higher at 90° tilting position. With the critical pressure application at 60 mmHg, the risk area was wider at 90° tilting compared to 30° tilting at each time point, but with no difference over time. In the case of 90° tilting, it is the result of the high pressure concentrated in the contact area around the great trochanter from the beginning. However, the 30° tilting position seems to maintain a relatively stable pressure owing to the effect that the pressure is spread evenly across the contact area rather than in the 90° tilting position.

Additionally, when comparing the time to reach 60 mmHg in the laterally inclined position, the 90° tilting was 21.5 min, which was noticeably shorter compared to the 78 min in the 30° tilting. Thus, a patient who changes position every 2 h was exposed to high pressure of 60 mmHg for about 100 min. Considering the studies that found that tissue changes could occur if pressure above 45–80 mmHg is continuously applied for more than an hour [20] and that the impact is minimal if it is less than an hour [19], the 90° tilting should be avoided as much as possible. If it is unavoidable to take a 90° tilting position, patients needed to change position at least every 1.5 h. However, when it is unavoidable to maintain a 90° tilting position for a long time, such as in surgery, additional research needs to confirm the effect of a support surface or preventive dressing that can be selected as an intervention to prevent pressure ulcers. However, if the participants of the 30° tilting position changed position every 2 h, the participant was exposed to a pressure of 60 mmHg or more for only 42 min in the immobile state; therefore, it can be a basis for maintaining the previously recommended period of position change of two hours. Even extending the position change interval can be considered carefully.

In the supine position, no significant difference showed in the scapula area over time or between 0° and 30° of head elevation, but a difference according to the time flow in the sacrum area occurred. Owing to the mechanism of intensive pressure accumulation in the sacrum region due to the generation of shear force when the head is raised [23], high pressure in the sacral region was expected to occur during the 30° HOB elevation supine. However, this region showed a risk of pressure ulcers regardless of whether the head rose or not. Therefore, preventive intervention is necessary. Even in the peak pressure results measured on the entire body interface, in the 30° HOB elevation supine position, measurements were more than 60 mmHg from the starting point, and higher pressure was observed at 30° HOB elevation supine than 0° HOB at all time points, although there was no statistically significant difference. As such, it was difficult to conclude a better position for pressure ulcers prevention in 0° and 30° HOB elevation supine position, similar to the results of a prior study [33] in which no significant difference showed in peak pressure between 0°, 10° and 30° HOB elevation supine.

There was no significant difference in the time to reach 60 mmHg between the supine position groups, with 40 and 26 min in a supine position with 0° and 30° HOB elevation respectively, both of which were less than 40 min. If the position is changed every 2 h, it means that patients are exposed to a pressure of more than 60 mmHg for about 80 min in a supine position with 0° HOB, and for 94 min in a supine position with 30° HOB elevation. Therefore, it is necessary to change the posture at least every 1.5 to 2 h and every 1.5 h for 0° and 30° HOB elevation supine position respectively.

In clinical practice, the supine position is the most common, and patients often have to raise their head above 30° owing to respiratory problems such as aspiration or pneumonia [13] or increased intracranial pressure [34]. Therefore, it is difficult to exclude the supine position altogether or to significantly reduce the time for changing the position to prevent pressure ulcers. Therefore, methods to maintain the pressure in the supine position at an appropriate level should be sought. For example, it may be considered to apply a position [33] such as angles of HOB elevation with the entire bed in the reverse Trendelenburg position, which shows lower pressure than when only raising the head. Additionally, the curvilinear supine position is reportedly effective in lowering pressure by evenly distributing the pressure across the entire body surface [35]. Thus, this may be a position to prevent bedsores. In combining and applying this modified supine position and additional preventive interventions, it can be effective in preventing pressure sores without shortening the time for position change; however, stronger evidence through additional research should be presented.

In Fowler’s position, the peak pressure measured in the scapula and sacrum showed a difference in time and no difference between the 30° and 45° HOB elevation Fowler’s groups. This result contradicts the existing guideline [9] that the head should be kept below 30°. Since there was no difference between 30° and 45°, patients who need to raise their heads do not need to keep their heads below 30°, and up to a 45° HOB elevation may be permitted. The time to reach 60 mmHg was 29 min for 30° of head elevation and 28.5 min for half sitting at 45° head elevation with no significant difference between the two groups. In a Fowler’s position, one might consider choosing the appropriate body position based on the clinical situation or the safety of the patients rather than the angle of raising the head.

However, it took only 30 min to reach a pressure of 60 mmHg or more in both the 30° and 45° HOB elevation Fowler’s positions; therefore, it is necessary to change the position at least every 1.5 h. The risk areas need to be observed more extensively. As in the supine position, a complementary intervention for preventing pressure ulcers should be considered, and in Fowler’s position, it is necessary to observe the risk area more extensively for pressure ulcers. In a previous study, a Fowler’s position between 15° and 45° HOB caused most pressure ulcers on the back [36], and in this study, we observed the highest pressure in the scapula at 120 min of 30° HOB elevation Fowler’s position. However, except for the 120-min time point in the 30° HOB elevation Fowler’s position, it was difficult to identify the risk area for pressure ulcers because higher pressure occurred in the sacrum rather than in the scapula at all time points in the 30° and 45° HOB elevation Fowler’s positions. Predicting the site of pressure ulcers is also particularly important as a strategy for preventing pressure ulcers, so it is necessary to reconfirm the site where the highest pressure occurs in Fowler’s position through repeated studies.

Combining the results of this study, when maintaining an immobile posture, the risk range for pressure sores increases over time, and it necessitates the adjustment of position change time according to each position. In particular, the 90° tilting position was high at the peak pressure, risk area ratio (at each reference point 30 mmHg, 60 mmHg) for each time period, and was confirmed as a position to be avoided for patients with a considerable risk of pressure ulcers. A 30° tilting position can be considered as the most stable position to prevent pressure ulcers by maintaining low pressure. Furthermore, the Fowler’s position, which raises the lower extremities together when the head is raised, is accepted as effective in preventing pressure ulcers in previous studies, but these results should be interpreted as limited to the sacrum region. Thus, the time to reach 60 mmHg within 30 min was short. With the inclusion of the entire contact area, it showed a high pressure of over 60 mmHg, and the time to reach 60 mmHg within 30 min was short.

The limitations of this study are that the results in healthy adults may differ from patient conditions and may not reflect pressure when using the support surface. Although this study measured the pressure of healthy adults on standard mattresses, since the reported interface pressure data so far are insufficient, this study is important in that they provide interface pressures in different positions and long-term cumulative pressures, as in real clinical practice.

## 6. Conclusions

The 90° body tilting position should be avoided owing to the increased risk of developing pressure and high pressure over time. However, the 30° tilting position effectively dispersed the pressure, and when exposed to more than 60 mmHg in a two-hour floating position, it was 42 min, the shortest period for the six body positions. This result supports existing guidelines that 30° tilting measures are effective in preventing pressure ulcers. If the 90° tilting position is inevitable, high pressure is concentrated in the greater trochanter, so preventive intervention is needed to prevent the occurrence of pressure ulcers in the greater trochanter area. No significant difference was observed in body interface pressure between the 0° and 30° HOB elevation supine, all of which produced the highest pressure in the sacral region. The 30° and 45° HOB elevation fowler also did not have a significant difference in interface pressure. Therefore, the result contrasted with the existing guideline that restricts head elevation of more than 30°. However, in the Fowler’s position, a wide range of skin assessments including the back is necessary because the peak pressure is not limited to the sacrum region. As pressure and time are not the only causes of pressure ulcers, we suggest a follow-up study that comprehensively considers various variables such as the support surface and the patient’s basal condition.

## Figures and Tables

**Table 1:** General characteristics of study participants.

Group	Categories	Mean ± SD ^1^ or *N* (%)		t or x^2^	*p*
		A	B		
Laterally inclined A: 30° tilting (*n* = 11) B: 90° tilting (*n* = 10)	Male	8 (80.8)	6 (54.5)	3.6	0.058
	Female	2 (20.0)	5 (45.5)	0.091	0.763
	Age (year)	24.00 ± 2.87	24.27 ± 2.00	−0.26	0.802
	BMI ^2^	23.10 ± 1.86	21.52 ± 2.76	1.52	0.145
Supine A: 0° HOB ^3^ (*n* = 11) B: 30° HOB ^3^ (*n* = 11)	Male	6 (54.5)	4 (36.4)	0.09	0.76
	Female	5 (45.5)	7 (63.6)	0.82	0.366
	Age (year)	22.91 ± 1.87	22.64 ± 2.34	0.30	0.765
	BMI ^2^	20.47 ± 2.12	21.48 ± 3.10	−0.8	0.385
Fowler’s A: 30° HOB ^3^ (*n* = 11) B: 45° HOB ^3^ (*n* = 11)	Male	5 (45.5)	3 (30.0)	0.09	0.763
	Female	6 (54.5)	7 (70.0)	0.16	0.206
	Age (year)	24.73 ± 1.90	23.20 ± 1.69	1.94	0.068
	BMI ^2^	22.22 ± 3.09	20.27 ± 2.40	1.60	0.127

^1^ Standard deviation; ^2^ Body mass index; ^3^ Head of bed.

**Table 2:** Comparison of peak pressure and risk area ratio between lateral positions with 30° and 90° tilting (*N* = 21).

Categories		Group	Baseline	30 min	60 min	90 min	120 min	Time	Group	T*G ^3^	X^2^(*p*)
			Mean ± SD ^1^ or Median (IQR ^2^)					F(*p*)			
Peak pressure	Shoulder	30°	42.84 ± 4.44	44.58 ± 3.20	44.97 ± 5.38	44.84 ± 6.36	46.93 ± 6.42	3.631 (0.029)	9.083 (0.007)	0.856 (0.445)	
		90°	55.82 ± 12.58	55.14 ± 13.35	55.94 ± 13.85	55.76 ± 9.98	62.41 ± 18.49				
		t(*p*)	−3.09 (0.010)	−2.44 (0.035)	−2.35 (0.038)	−3.02 (0.007)	−2.51 (0.029)				
	Greater trochanter	30°	47.45 ± 11.53	49.55 ± 13.59	49.87 ± 13.94	50.84 ± 14.88	50.85 ± 12.52	6.350 (0.002)	13.446 (0.002)	2.473 (0.082)	
		90°	69.54 ± 19.05	74.00 ± 19.63	80.73 ± 26.68	81.34 ± 21.03	79.15 ± 20.61				
		t(*p*)	−3.18 (0.006)	−3.35 (0.005)	−3.37 (0.003)	−3.87 (0.001)	−3.76 (0.002)				
	(Total area)	30°	50.13 ± 11.10	51.28 ± 12.79	57.96 ± 15.94	54.54 ± 16.69	54.32 ± 15.94				
		90°	72.30 ± 16.89	76.86 ± 18.03	82.26 ± 25.23	81.85 ± 20.36	82.84 ± 18.97				
Risk area ratio	IP ^4^ ≥ 30 mmHg	30°	25.69 ± 4.76	27.91 ± 4.72	28.73 ± 5.67	29.50 ± 5.57	29.59 ± 5.00	27.032 (<0.001)	16.724 (0.001)	2.928 (0.026)	
		90°	34.00 ± 5.51	36.20 ± 6.30	39.10 ± 6.78	39.48 ± 6.83	41.32 ± 5.93				
		t(*p*)	−3.71 (0.001)	−3.43 (0.003)	−3.82 (0.001)	−3.68 (0.002)	−4.92 (<0.001)				
	IP ^4^ ≥ 60 mmHg	30°	0.00 (0.00)	0.00 (0.00)	0.00 (0.00)	0.00 (0.00)	0.00 (0.00)				8.42 (0.077)
		90°	1.00 (2.07)	1.38 (1.98)	1.52 (1.48)	1.56 (1.54)	1.85 (2.51)				4.52 (0.341)
		Z(*p*)	−2.61 (0.009)	−3.04 (0.002)	−2.76 (0.006)	−2.81 (0.005)	−3.17 (0.002)				

^1^ Standard deviation; ^2^ Interquartile Range; ^3^ Time*Group; ^4^ Interface Pressure.

**Table 3:** Comparison of peak pressure and risk area ratio between a supine position with 0° and 30 ° HOB elevation (*N* = 22).

Categories		Group	Baseline	30 min	60 min	90 min	120 min	Time	Group	T*G ^3^	X^2^(*p*)
			Mean ± SD ^1^ or Median (IQR ^2^)					F(*p*)			
Peak pressure	Scapula	0°	41.56 ± 17.18	48.11 ± 12.26	50.13 ± 11.25	46.71 ± 5.74	50.46 ± 9.49	2.807 (0.063)	0.246 (0.625)	0.235 (0.824)	
		30°	46.23 ± 10.62	50.88 ± 14.14	52.12 ± 16.42	47.11 ± 9.44	51.03 ± 13.12				
	Sacrum	0°	52.41 ± 3.89	57.95 ± 6.98	57.88 ± 7.56	62.82 ± 9.76	65.05 ± 8.95	30.677 (<0.001)	2.147 (0.158)	0.595 (0.590)	
		30°	56.47 ± 8.40	62.44 ± 10.17	65.53 ± 10.83	67.78 ± 10.48	70.03 ± 12.24				
	(Total area)	0°	54.87 ± 7.11	61.06 ± 9.71	61.09 ± 9.63	67.06 ± 17.66	66.13 ± 7.53				
		30°	60.35 ± 9.25	65.87 ± 9.05	66.80 ± 12.06	67.78 ± 10.48	71.51 ± 11.53				
Risk area ratio	IP ^4^ ≥ 30 mmHg	0°	28.75 ± 6.75	32.86 ± 6.07	34.07 ± 4.12	34.85 ± 5.03	34.68 ± 5.15	0.716 (<0.001)	1.002 (0.329)	0.404 (0.805)	
		30°	27.78 ± 4.28	30.57 ± 4.67	31.64 ± 5.40	32.43 ± 5.72	32.33 ± 5.49				
	IP ^4^ ≥ 60 mmHg	0°	0.00 (0.00)	0.00 (0.44)	0.00 (0.43)	0.41 (0.96)	0.79 (2.52)				11.03 (0.026)
		30°	0.00 (0.96)	1.11 (1.75)	1.06 (2.79)	1.61 (2.22)	1.42 (2.51)				19.31 (<0.001)
		Z(*p*)	−1.47 (0.142)	−2.14 (0.032)	−2.14 (0.032)	−2.10 (0.036)	−1.91 (0.056)				

^1^ Standard deviation; ^2^ Interquartile Range; ^3^ Time*Group; ^4^ Interface Pressure.

**Table 4:** Comparison of peak pressure and risk area ratio between Fowler’s positions with 30° and 45° HOB (*N* = 22).

Categories		Group	Baseline	30 min	60 min	90 min	120 min	Time	Group	T*G ^3^	X^2^(*p*)
			Mean ± SD ^1^ or Median (IQR ^2^)					F(*p*)			
Peak pressure	Scapula	30°	50.66 ± 8.27	53.19 ± 10.54	54.94 ± 7.94	58.80 ± 9.10	60.10 ± 11.13	5.404 (0.001)	0.002 (0.964)	0.854 (0.496)	
		45°	51.32 ± 11.65	56.87 ± 11.64	55.84 ± 9.57	56.31 ± 11.06	58.26 ± 13.38				
	Sacrum	30°	55.19 ± 12.39	58.62 ± 14.39	58.76 ± 13.45	58.29 ± 13.56	58.06 ± 14.10	7.954 (0.002)	1.244 (0.279)	2.796 (0.083)	
		45°	60.65 ± 18.96	64.31 ± 20.77	66.59 ± 22.40	68.21 ± 19.29	70.24 ± 20.92				
	(Total area)	30°	59.80 ± 11.96	66.58 ± 16.06	67.45 ± 14.95	65.63 ± 15.11	66.03 ± 15.17				
		45°	66.73 ± 16.76	72.71 ± 19.86	74.03 ± 21.73	73.79 ± 17.77	79.65 ± 18.67				
Risk area ratio	IP ^4^ ≥ 30 mmHg	30°	26.25 ± 5.74	28.46 ± 6.62	29.90 ± 6.18	30.37 ± 5.86	31.72 ± 6.11	20.020 (<0.001)	0.716 (0.408)	1.395 (0.257)	
		45°	29.24 ± 4.65	31.06 ± 4.47	31.38 ± 4.75	32.12 ± 4.61	32.51 ± 4.11				
	IP ^4^ ≥ 60 mmHg	30°	0.30 (0.52)	0.34 (1.38)	0.51 (1.02)	0.70 (1.27)	0.76 (1.63)				3.29 (0.510)
		45°	0.39 (0.59)	0.75 (0.77)	0.70 (1.04)	0.60 (1.14)	1.04 (0.94)				6.47 (0.167)
		Z(*p*)	−0.69 (0.491)	−0.18 (0.858)	−0.18 (0.859)	−0.36 (0.722)	−0.21 (0.831)				

^1^ Standard deviation; ^2^ Interquartile Range; ^3^ Time*Group; ^4^ Interface Pressure.

## Data Availability

The data presented in this study are available on request from the corresponding author.
